# Classical conditioning without verbal suggestions elicits placebo analgesia and nocebo hyperalgesia

**DOI:** 10.1371/journal.pone.0181856

**Published:** 2017-07-27

**Authors:** Przemysław Bąbel, Elżbieta A. Bajcar, Wacław Adamczyk, Paweł Kicman, Natalia Lisińska, Karolina Świder, Luana Colloca

**Affiliations:** 1 Pain Research Group, Institute of Psychology, Jagiellonian University, Kraków, Poland; 2 Department of Kinesiotherapy and Special Methods in Physiotherapy, The Jerzy Kukuczka Academy of Physical Education, Katowice, Poland; 3 Donders Institute for Brain, Cognition & Behaviour, Radboud University, Nijmegen, The Netherlands; 4 Department of Pain and Translational Symptom Science, University of Maryland School of Nursing, Baltimore, Maryland, United States of America; 5 Department of Anesthesiology, School of Medicine, University of Maryland School of Nursing, Baltimore, Maryland, United States of America; University of Bologna, ITALY

## Abstract

The aim of this study was to examine the relationships among classical conditioning, expectancy, and fear in placebo analgesia and nocebo hyperalgesia. A total of 42 healthy volunteers were randomly assigned to three groups: placebo, nocebo, and control. They received 96 electrical stimuli, preceded by either orange or blue lights. A hidden conditioning procedure, in which participants were not informed about the meaning of coloured lights, was performed in the placebo and nocebo groups. Light of one colour was paired with pain stimuli of moderate intensity (control stimuli), and light of the other colour was paired with either nonpainful stimuli (in the placebo group) or painful stimuli of high intensity (in the nocebo group). In the control group, both colour lights were followed by control stimuli of moderate intensity without any conditioning procedure. Participants rated pain intensity, expectancy of pain intensity, and fear. In the testing phase, when both of the coloured lights were followed by identical moderate pain stimuli, we found a significant analgesic effect in the placebo group, and a significant hyperalgesic effect in the nocebo group. Neither expectancy nor fear ratings predicted placebo analgesia or nocebo hyperalgesia. It appears that a hidden conditioning procedure, without any explicit verbal suggestions, elicits placebo and nocebo effects, however we found no evidence that these effects are predicted by either expectancy or fear. These results suggest that classical conditioning may be a distinct mechanism for placebo and nocebo effects.

## Introduction

There is a growing body of evidence that classical conditioning can enhance placebo analgesia induced by verbal suggestions [1–4] and that the effects of classical conditioning on placebo analgesia induced by verbal suggestions are likely to be mediated by expectancies [5,6]. The concept of expectancy or expectation is understood to mean a ‘conscious, conceptual belief about the future occurrence of an event’ [7, p. 406]. Moreover, it has been found that classical conditioning may enhance the expectancies induced by verbal suggestions even if they do not enhance placebo analgesia [8]. The placebo effects induced by classical conditioning along with verbal suggestions persist even when expectancies are miminised by revealing the true nature of the treatment, i.e. that it does not have pain-relieving properties [9].

However, little is known about the role of expectancy in placebo analgesia induced by classical conditioning without explicit verbal suggestions. A few studies have attempted to induce placebo analgesia by using classical conditioning alone [1,10–13], and most of them succeeded [1,11–13]. Although these results strongly support the idea that classical conditioning is not necessarily mediated by expectancy, participants were explicitly asked to rate their expectancies in only two of the these studies [11,13]. Indeed, in most of the previous studies on placebo effects, expectancy of pain intensity was not rated [1,3,4,10,12,14–17], and studies in which it was rated included clear verbal suggestions [2,5,6,9,13,18–20].

Although previous attempts to induce nocebo hyperalgesia by classical conditioning without verbal suggestions have failed [21], the effects of conditioning and verbal suggestions on nocebo hyperalgesia have been found not to be greater than the effects of verbal suggestions alone [21,22]. These results suggest that conditioning, per se, does not significantly increase the nocebo effects induced by verbal suggestions alone. Moreover, recent findings on placebo analgesia and nocebo hyperalgesia induced by subliminal cues suggest that the effects of conditioning may not necessarily involve expectancy changes [23,24].

The first aim of the study was to induce placebo analgesia and nocebo hyperalgesia using a hidden conditioning procedure that did not include any explicit verbal suggestions about the possibility of experiencing less or more intensity of pain. Our second aim was to investigate to what extent self-reported expectancy of pain intensity can predict the placebo and nocebo effects induced by classical conditioning alone, without providing participants with any expectation of benefit or harm or any information related to the meaning of the cues. We hypothesised that when classical conditioning is used to induce placebo effects without any verbal suggestions, expectancy is not critically involved in eliciting placebo and nocebo effects. The final aim of the study was to determine the role of fear in shaping placebo and nocebo effects. Although it has been shown that fear and stress may eliminate placebo effects induced by verbal suggestions [25], it remains to be established what the role of fear is in the formation of placebo analgesia and nocebo hyperalgesia induced by classical conditioning in the absence of explicit verbal suggestions.

## Material and methods

### Participants

A total of 42 female volunteers (mean age = 20.83 ± 1.46, range = 18–27 years) participated in the study. They were randomly assigned to three groups: placebo, nocebo and control groups. Sample size was based on previous studies that used a similar ‘n’ (14 persons in each group) and had shown significant effects in similar paradigms [15–17,22,26] (see Table 1). All of the participants were healthy, free of pain and did not take any type of pain medication; none of them had any contraindications for electrical stimulation and none of them had previously participated in any pain-related studies. Participants were informed that the aim of the study was to investigate responses to electrical stimulation and that they would receive a series of electrical stimuli during the study. They were also informed that they could stop participating at any point during the study without providing a reason for their withdrawal. After having read the description of the study’s procedure, participants gave their informed written consent to participate in the experiment. The study protocol was approved by the Research Ethics Committee at the Institute of Psychology of Jagiellonian University.

**Table 1:** Characteristics of the subjects in each experimental group.

Group	N	Sex	Age	BMI	*t*	*T*
Placebo	14	F	20.21 ± 1.25	19.56 ± 2.57	2.16 ± 0.91	13.65 ± 12.46
Nocebo	14	F	21.64 ± 1.78	22.70 ± 3.57	2.09 ± 0.98	16.86 ± 18.83
Control	14	F	20.64 ± 1.01	20.53 ±1.27	2.05 ± 0.57	16.59 ± 16.04

N = number of participants in each group.

F = female.

BMI = body mass index.

*t* = nonpainful tactile.

*T* = pain threshold.

### Stimuli

The stimuli were electric shocks delivered to the volar surface of the nondominant forearm through two durable stainless steel-disk electrodes 8 mm in diameter with 30 mm spacing. The electrical stimuli were square pulses with a duration of 200 μs delivered by the Constant Current High Voltage Stimulator (Digitimer, Welwyn Garden City, England, model DS7AH). The intensity of the electrical stimuli was set up individually for each participant according to a calibration procedure (see below) in which the level of nonpainful tactile sensation (*t*) and the pain threshold (*T*) were determined. Depending on the experimental group, the intensity of the electrical stimuli (expressed in mA) was set at either [*t* + 0.8 × (*T—t*)] mA (paired with placebo stimuli), or (2.2 × *T*—0.2 × *t*) mA (paired with nocebo stimuli). The formula [*t* + 0.8 × (*T—t*)] was used to ensure that the stimulus resulted in a clear tactile but nonpainful sensation. The coefficient 0.8 was established on the basis of the results of a preliminary study. The formula for electrical stimuli paired with nocebo stimuli (2.2 × *T*—0.2 × *t*) was established so that the calculated value was higher than the value of the control stimuli in the same proportion as the stimuli paired with placebo stimuli was lower with respect to the control stimuli. Regardless of group assignment, stimuli of the intensity of 1.5 × *T* mA served as control stimuli. All participants received a total of 96 stimuli, excluding the calibration phase.

The electrical stimuli were preceded by the presentation of light stimuli presented in full-screen mode on a computer screen (17", resolution 1280 x 1024) facing the subject at a distance of approximately 50 cm. Two colours of light stimuli were used–blue and orange. Either the blue or orange colour acted as a placebo/nocebo stimulus. The colours of the light stimuli were counterbalanced in two ways. For half of the participants, the blue colour was a placebo/nocebo stimulus when it preceded a less/more painful stimulus and the orange colour was a control stimulus. For the remaining half of the participants, the colours of the light stimuli were reversed (blue = control stimulus; orange = placebo/nocebo stimulus). Half of the participants started with the blue light, and the other half started with the orange light as the first light presented on the computer screen.

### Measures

The participants rated pain intensity, expectancy of pain intensity, and fear by means of an 11-point numeric rating scale (NRS). The scales for pain intensity and expectancy of pain intensity ratings ranged from 0 = ‘no pain’ to 10 = ‘the most pain that is tolerable’. Fear was rated on a scale ranging from 0 = ‘not at all’ to 10 = ‘very much’. At the end of the experiment all participants were asked to answer a question designed to determine if they had deciphered the actual aim of the study. However, nobody did decipher the actual aim of the study.

### Design and procedures

**Fig 1:**
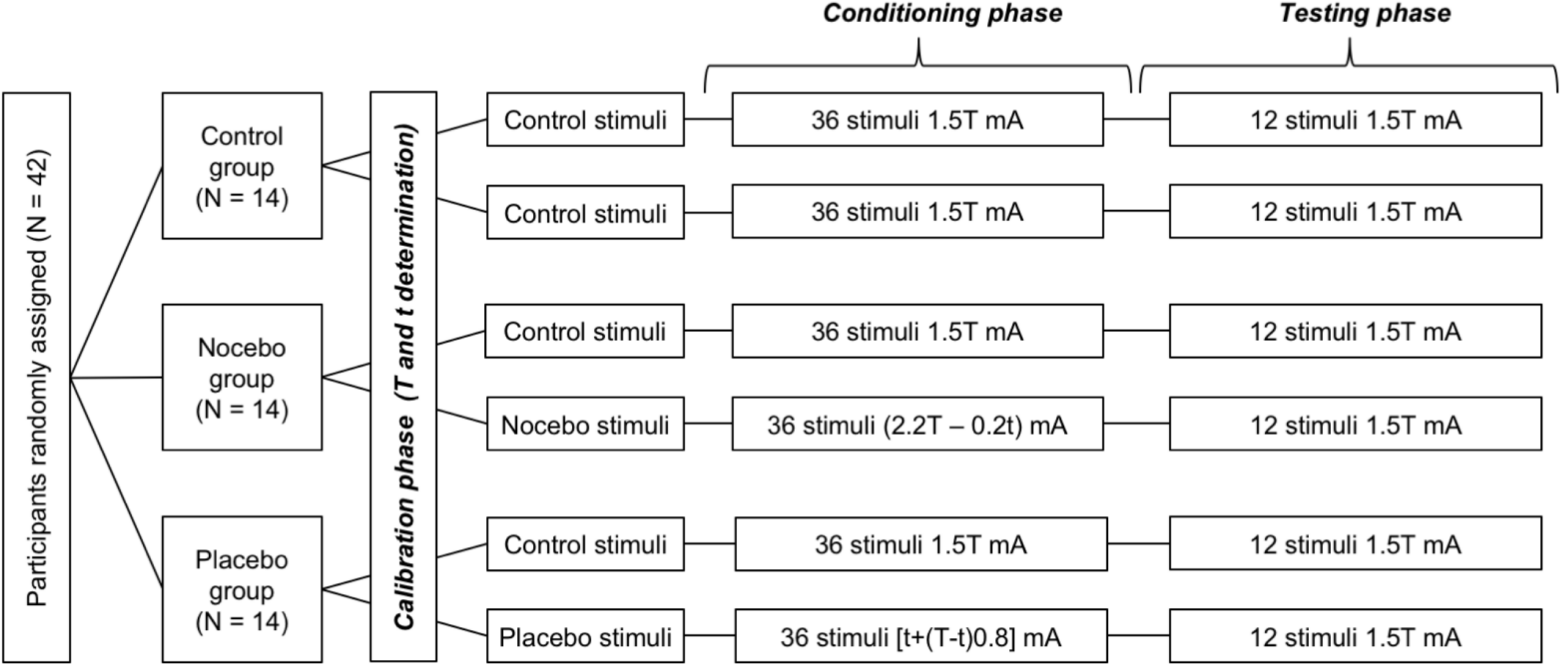
Study design. A total of 42 female volunteers were randomly assigned to three groups: placebo, nocebo, and control group. Each group consisted of 14 participants. The study consisted of three phases: calibration, conditioning, and testing. Calibration was conducted to determine nonpainful tactile sensation (*t*) and the pain threshold (*T*). In the conditioning phase, a total of 72 electrical stimuli were delivered–half of the stimuli were of moderate intensity (1.5 × *T* mA, control stimuli) and the remaining half were nonpainful ([*t* + 0.8 × (*T—t*)] mA in placebo group) or higher intensity ([2.2 × *T*—0.2 × *t*] mA in nocebo group). The intensity of the electrical stimuli was always set at 1.5 × T mA in the control group. The testing phase consisted of 24 control stimuli (1.5 × T mA) regardless of group assignment.

The study consisted of three phases: calibration, conditioning, and testing (see Fig 1).

#### Calibration phase

Calibration was conducted to determine the intensity of both painful stimuli (paired with nocebo stimuli) and nonpainful stimuli (paired with placebo stimuli) individually. The calibration procedure was based on the method of limits applied in previous studies in which electrical stimulation was used to induce pain [17,26]. First, *t* and *T* were determined as follows. Ascending series of stimuli in steps of 0.5 mA (the interstimulus interval was 5 sec) were delivered starting at 0 mA. The intensity of the electrical stimuli was gradually increased until participants detected their first nonpainful tactile sensation (*t*). The intensity was further increased until the detected sensations became painful (*T*), which was clearly stated verbally by the participant. Then, the averaged values for *t* and *T* were calculated in order to determine the stimulus intensity for the conditioning procedure.

#### Conditioning phase

The conditioning phase was started five minutes after the calibration phase was completed. A total of 72 electrical stimuli, divided into 4 blocks (18 stimuli each) with a 2 minute break between blocks, were delivered in a pseudorandom sequence–half of the stimuli in each block were of moderate intensity (1.5 × T mA, control stimuli) and the remaining half were nonpainful ([*t* + 0.8 × (*T—t*)] mA in the placebo group) or higher intensity ([2.2 × *T*—0.2 × *t*] mA in the nocebo group). An electrical stimulus lasting 200 μs was delivered during the presentation of a black background, which was displayed for 2 sec. Before each electrical stimulus was applied, a blue or orange light was shown in a pseudorandom sequence for 10 sec. One of the colours was paired with control stimuli and the other with nonpainful stimuli (in the placebo group) or more painful stimuli (in the nocebo group). Participants were not informed about that association.

**Fig 2:**
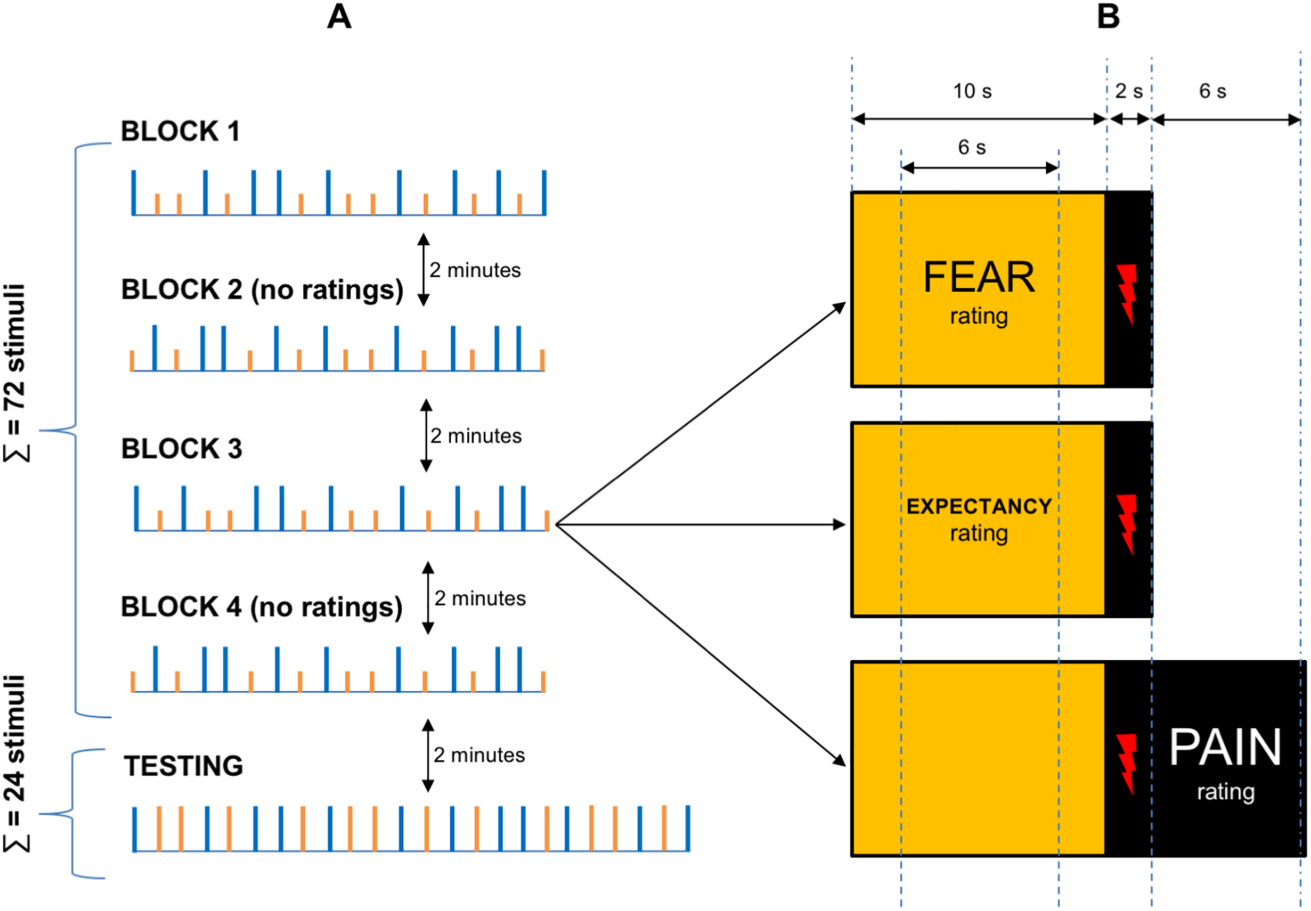
Details of the study design using an example of the placebo group with an orange light serving as a placebo. Part ‘A’ depicts the time-course of the procedure: there were four blocks of conditioning trials, two of them with pain, expectancy, and fear ratings (Blocks 1 and 3), and two without any ratings (Blocks 2 and 4). Each conditioning block consisted of 18 electrical stimuli. After the conditioning phase was completed, the testing phase consisting of 24 electrical stimuli began. Orange lights (orange vertical bars) served as placebo stimuli (nonpainful intensity, i.e. [*t* + 0.8 × (*T—t*)] mA), while blue lights served as control stimuli (painful intensity, i.e. 1.5 × T mA). During the testing phase, the stimuli of the same painful intensity (i.e. 1.5 × T mA) were applied, regardless of the colour of the preceding light. Part ‘B’ depicts the design of single a trial: a colour light was presented for 10 seconds. For each block, one-third of the lights was displayed with the NRS for the expectancy rating, one-third was displayed with the NRS for the fear rating and one-third was displayed without any scale. In the latter case, a slide with the NRS for pain intensity rating was shown for another 6 sec immediately after the electrical stimulus (depicted by red lightning) was applied.

The participants rated pain intensity, expectancy of pain intensity, and fear during the first and the third blocks of stimuli. The NRS for expectancy and fear ratings were shown during the presentation of the light stimuli. The NRS was shown 2 sec after the presentation of the light alone and lasted for 6 sec, followed by 2 sec of light alone. When 10 sec of light presentation was completed, a black background was presented and the electrical stimulus was delivered. For each block, one-third of the lights was displayed with the NRS for the expectancy of pain intensity rating, one-third was displayed with the NRS for the fear rating and one-third was displayed without any scale. In the latter case, a slide with the NRS for pain intensity rating was shown for another 6 sec immediately after the electrical stimulus was applied (see Fig 2).

Participants did not rate pain intensity, expectancy of pain intensity or fear during the second and the fourth blocks of stimuli. After 10 sec of light presentation (without any additional information on the slide), the black background was presented and the electrical stimulus was delivered.

#### Testing phase

The testing phase began two minutes after the conditioning phase was completed. It consisted of 24 control stimuli (1.5 × T mA) preceded by 12 orange and 12 blue light stimuli presented in a pseudorandom sequence. The pain intensity, expectancy of pain intensity, and fear ratings were performed in the same way they were during the first and the third blocks of the conditioning phase, with one exception–pain was rated 12 times, while expectancy was rated 6 times and fear was rated 6 times.

The procedure in the control group was similar to that applied in the experimental groups. The only difference was that the intensities of the electrical stimuli were always set at 1.5 × T mA, regardless of the colour of the light stimuli (blue or orange). Participants were not aware that all the stimuli were set at the same level. Pain intensity, expectancy of pain intensity, and fear were rated in the same way as they were in the placebo and nocebo groups. Applying only one level of stimuli, i.e. 1.5 × T mA allowed to control for nonassociative learning effects (sensitisation and habituation) and the effects of colours on pain perception. Such designed control groups were used in previous studies in which placebo analgesia was induced by classical conditioning [26,27].

### Statistical analysis

#### Manipulation check

To control for baseline differences in pain, NRS pain ratings from conditioning phase of the study were compared using a repeated-measures analysis of variance (ANOVA) design, with experimental group (placebo, nocebo, and control group) as a between-subject factor and condition (placebo/nocebo- and control-associated NRS ratings) as a within-subject factor. The *F*-tests were followed by *post-hoc* comparisons for manipulation check. Differences between placebo versus control stimuli (placebo group), nocebo versus control stimuli (nocebo group), and blue-control versus orange-control stimuli (control group) were tested in *post-hoc* comparisons.

To explore internal validity of the results, predictive validity of expectancy was evaluated. Pearson correlation coefficients (*r*) between expectancy of one type of stimuli (e.g. placebo, nocebo or control) and pain intensity associated with the same stimuli were calculated. To investigate reliability of the measurement, intraclass correlation coefficients (ICC_(3,3–6)_) were calculated based on repeated measurements of each variable: pain intensity, expectancy, and fear. ICCs values were calculated for each conditioning and testing blocks separately. ICCs above 0.75 were considered as a good reliability level [28].

#### Induction of the placebo analgesia and nocebo hyperalgesia

In order to verify hypotheses, data from testing phase of the study was analysed. Statistical comparisons were performed using a repeated-measures ANOVA design, with experimental group (placebo, nocebo, and control group) as a between-subject factor and condition (placebo/nocebo- and control-associated NRS ratings) as a within-subject factor. Separate ANOVAs were conducted for each dependent variable: pain intensity, expectancy of pain intensity, and fear in the testing phase of the study. In order to test whether conditioning was effective, the *F*-tests were followed by within-group planned-comparison tests: (1) placebo- versus control-associated NRS ratings in the placebo group, (2) nocebo- versus control-associated NRS ratings in the nocebo group, and (3) blue-control- versus orange-control-associated NRS ratings in the control group. Separate comparisons were conducted for NRS ratings of: (a) pain intensity, (b) expectancy of pain intensity, and (c) fear.

In the next step of the analyses, between-group planned-comparison tests were performed. To determine whether the placebo analgesia was induced, the mean difference in NRS pain ratings between placebo and control stimuli from the placebo group was compared to the mean difference between two control (blue and orange) stimuli from the control group. Similarly, to determine whether the nocebo hyperalgesia was induced, the mean difference in NRS pain ratings between nocebo and control stimuli from the nocebo group was compared to the mean difference between two control (blue and orange) stimuli from the control group. Similar comparisons were conducted for NRS ratings of expectancy of pain intensity and fear.

#### Regression analyses

Forward, stepwise multiple regression was performed to determine the degree to which the mean difference in pain intensity of the placebo/nocebo- and control-associated stimuli was predicted by (1) the mean difference in expectancies for placebo/nocebo- and control-associated stimuli, and (2) the mean difference in fear for placebo/nocebo- and control-associated stimuli. Separate analyses were performed for each of the three groups for the testing phase. The two predictor (or independent) variables were tested in each of the three regression analyses.

All the analyses were conducted using the STATISTICA data analysis software, version 10 (StatSoft Inc., Tulsa, OK, USA), with the exception of the compromise power analyses and effect sizes calculations, which were performed using G*Power 3.1.9.2 [29,30]. The level of significance was set at *p* < 0.05 for rejecting the null hypothesis in all the statistical analyses.

## Results

### Manipulation check

ANOVA on the pain ratings from conditioning phase revealed a statistically significant interaction between experimental group and condition (*F*_(2, 39)_ = 41.90, *p* < 0.001, *ŋ*^2^ = 0.68). No significant main effects of experimental group or condition were found. *Post-hoc* tests revealed that there were no baseline differences in NRS pain ratings for control stimuli across experimental groups, indicating that pain produced by control stimuli was rated similarly among experimental groups. However, placebo stimuli were rated as less painful (1.04 ± 1.24) compared to control stimuli (3.38 ± 2.16) in the placebo group (p < 0.001) and nocebo stimuli (4.55 ± 2.04) were rated as more painful compared to control stimuli (2.98 ± 1.87) in the nocebo group (p < 0.001), indicating that participants discriminated between more and less painful stimuli in the placebo and nocebo group. In the control group, there was no difference in NRS pain ratings for control stimuli associated with one colour (e.g. orange, 2.88 ± 2.37) compared to control stimuli associated with other colour (e.g. blue, 2.80 ± 2.29).

Predictive validity of expectancy was very high. Expectancy of pain intensity associated with placebo stimuli was positively correlated with pain intensity experienced after placebo stimuli (*r* = 0.61, p < 0.05), and expectancy of pain intensity associated with control stimuli was positively correlated with pain intensity experienced after control stimuli (*r* = 0.85, *p* < 0.001) in the placebo group. Similar results were observed in case of the expectancy and pain ratings of nocebo (*r* = 0.80, *p* < 0.01) and control stimuli (*r* = 0.75, *p* < 0.01) in the nocebo group, and blue-control (*r* = 0.96, *p* < 0.001) and orange-control (*r* = 0.77, *p* < 0.01) stimuli in the control group.

Reliability coefficients for measured variables are presented in Table 2. Pain ratings were stable across conditioning and testing blocks and were characterized by excellent reliability level (ICC_(3,6)_ ≥ 0.90). Reliability of expectancy and fear ratings was low to moderate (ICC_(3,3)_ = 0.41–0.70) during first conditioning block, moderate to good during third conditioning block (ICC_(3,3)_ = 0.68–0.89) and good during testing block (ICC_(3,3)_ > 0.85).

**Table 2:** Reliability of measurement. Intraclass correlation coefficients for each of measured variables associated with condition stimuli (placebo, nocebo, control) or control stimuli.

Stimuli	Variable	Block 1		Block 3		Testing	
		ICC^c^	95% CI^d^	ICC^c^	95% CI^d^	ICC^c^	95% CI^d^
Condition^a^	Expectancy	0.44	0.25–0.62	0.89	0.82–0.93	0.85	0.76–0.91
	Pain	0.90	0.85–0.94	0.92	0.88–0.96	0.91	0.87–0.95
	Fear	0.51	0.32–0.67	0.92	0.88–0.96	0.88	0.82–0.93
Control^b^	Expectancy	0.41	0.22–0.60	0.68	0.53–0.80	0.89	0.82–0.93
	Pain	0.87	0.79–0.92	0.93	0.89–0.96	0.91	0.87–0.95
	Fear	0.70	0.56–0.81	0.88	0.81–0.93	0.88	0.81–0.93

^a^ Condition refers to stimuli that were associated with placebo (placebo group), nocebo (nocebo group) or control (control group).

^b^ Control refers to stimuli that served as control stimuli in each of the group.

^c^ Intraclass correlation coefficient.

^d^ Confidence intervals.

### Induction of the placebo analgesia and nocebo hyperalgesia

The descriptive statistics for all the analysed variables are presented in Table 3. ANOVA on the pain ratings from the testing phase of the study revealed a statistically significant interaction between experimental group and condition (*F*_(2, 39)_ = 26.17, *p* < 0.001, *ŋ*^2^ = 0.57). No significant main effects of experimental group or condition were found. Within-group planned comparison on placebo- versus control-associated NRS pain intensity ratings revealed a statistically significant difference for the placebo group (*F*_(1, 39)_ = 30.18, *p* < 0.001, *ŋ*^2^ = 0.44), indicating that the electrical stimuli associated with the placebo stimuli were rated as less painful than the control stimuli (mean difference 0.41 ± 0.19). Within-group planned comparison on nocebo- versus control- associated NRS pain intensity ratings revealed significant difference for the nocebo group (*F*_(1, 39)_ = 21.95, *p* < 0.001, *ŋ*^2^ = 0.36), indicating that the electrical stimuli associated with the nocebo stimuli were rated as more painful than the control stimuli (mean difference 0.35 ± 0.38). By contrast, no statistically significant difference was found between two control stimuli in the control group (mean difference 0.04 ± 0.22).

**Table 3:** Descriptive statistics for all the analyzed variables from the testing phase of the study (Mean ± SD).

Variable	Stimuli	Placebo group	Nocebo group	Control group^b^
Pain intensity	Placebo/nocebo	3.14 ± 2.38	2.98 ± 2.17	3.02 ± 2.76
	Control	3.55 ± 2.35	2.63 ± 2.08	2.99 ± 2.73
	Difference^a^	0.41 ± 0.19	-0.35 ± 0.38	-0.04 ± 0.22
Expectancy	Placebo/nocebo	3.07 ± 1.98	3.57 ± 2.57	3.10 ± 2.97
	Control	3.36 ± 2.13	3.14 ± 2.15	3.26 ± 2.94
	Difference^a^	0.29 ± 0.70	-0.43 ± 1.14	0.17 ± 0.50
Fear	Placebo/Nocebo	1.48 ± 1.56	2.67 ± 2.17	0.95 ± 1.25
	Control	1.64 ± 1.80	2.29 ± 1.91	1.05 ± 1.21
	Difference^a^	0.17 ± 0.69	-0.38 ± 1.12	0.10 ± 0.36

^a^ Difference between NRS ratings of placebo- or nocebo-associated stimuli.

^b^ In the control group, both stimuli were set at the same level of intensity; therefore, the differences presented here can be considered as the differences between the blue- and orange-associated NRS ratings.

**Fig 3:**
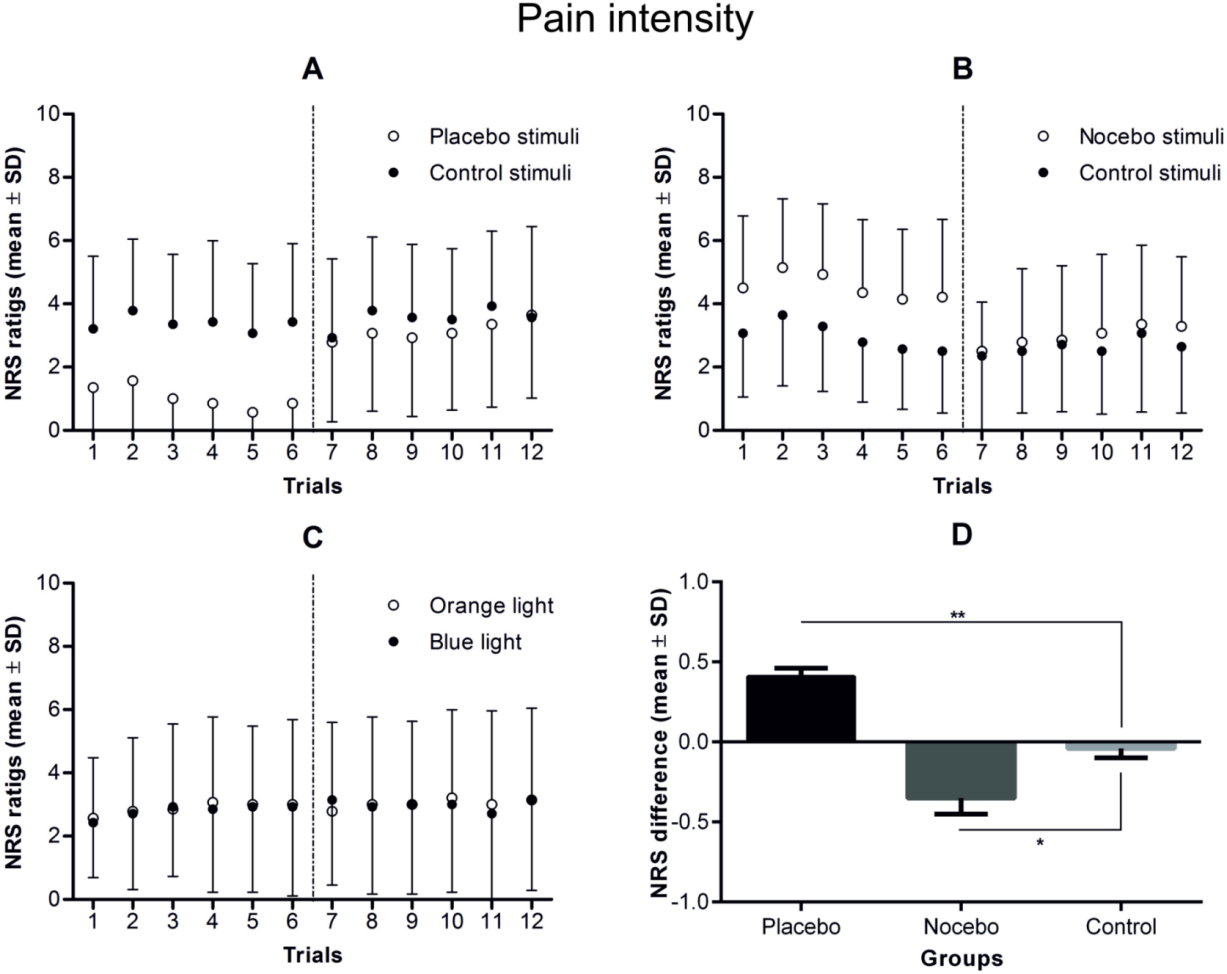
**Pain ratings during the conditioning and testing phases in placebo (A), nocebo (B) and control (C) group.** Note that although the conditioning phase consisted of 72 electrical stimuli, which were divided into 4 blocks of 18 stimuli, the participants rated pain intensity in only one-third of the trials during the first and the third blocks of stimuli. In the testing phase of the study (separated by vertical dotted lines), which consisted of 24 control stimuli, pain intensity was rated 12 times (6 for placebo/nocebo and 6 for control stimuli). The mean differences in pain intensity during the testing phase of the study in each of the study groups are presented in part D of the figure. In the testing phase, there were not only statistically significant differences in pain intensity within the placebo and nocebo groups, but the differences in pain intensity in the placebo and nocebo groups were significantly higher than in the control group. * *p* < 0.01, ** *p* < 0.001.

Between-group planned comparison on the difference between placebo- and control-associated NRS pain intensity ratings from the placebo group compared to the difference between blue-control- and orange-control-associated NRS pain intensity ratings from the control group revealed a statistically significant effect (*F*_(1, 39)_ = 17.87, *p* < 0.001, *ŋ*^2^ = 0.31), indicating that placebo analgesia was induced by classical conditioning. Between-group planned comparison on the difference between nocebo- and control-associated NRS pain intensity ratings from the nocebo group compared to the difference between blue-control- and orange-control-associated NRS pain intensity ratings from the control group revealed also a statistically significant difference (*F*_(1, 39)_ = 8.82, *p* < 0.01, *ŋ*^2^ = 0.18), indicating that nocebo hyperalgesia was induced by classical conditioning without any explicit information (Fig 3 and Table 4).

**Table 4:** The NRS results of repeated-measures analyses of variance (ANOVA) and planned comparisons.

Variable	Main effects and interactions	*F*	*df*	*p* *(power)*	*ƞ*^*2*^	Planned comparisons		*F*	*df*	*p* *(power)*	*ƞ*^*2*^
Pain intensity						Placebo group^a^	Placebo vs control ratings	30.18	1, 39	0.001 (0.99)	0.44
	Rating	0.04	1, 39	0.85 (0.08)	0.001	Nocebo group^a^	Nocebo vs control ratings	21.95	1, 39	0.001 (0.99)	0.36
	Experimental condition	0.18	2, 39	0.84 (0.20)	0.009	Control group^a^	Orange-control vs blue-control ratings	0.24	1, 39	0.63 (0.24)	0.01
	Experimental condition × Rating	26.17	2, 39	0.0001 (0.99)	0.57	Difference between placebo/nocebo vs control stimuli^b^	Placebo vs control group	17.87	1, 39	0.001 (0.99)	0.31
							Nocebo vs control group	8.82	1, 39	0.005 (0.99)	0.18
Expectancy						Placebo group^a^	Placebo vs control ratings	1.67	1, 39	0.20 (0.84)	0.04
	Rating	0.004	1, 39	0.95 (0.05)	0.001	Nocebo group^a^	Nocebo vs control ratings	3.76	1, 39	0.06 (0.78)	0.09
	Experimental condition	0.02	2, 39	0.98 (0.05)	0.001	Control group^a^	Orange-control vs blue-control ratings	0.56	1, 39	0.46 (0.70)	0.01
	Experimental condition × Rating	3.00	2, 39	0.0615 (0.99)	0.13	Difference between placebo/nocebo vs control stimuli^b^	Placebo vs control group	0.15	1, 39	0.71 (0.23)	0.01
							Nocebo vs control group	3.62	1, 39	0.06 (0.38)	0.09
Fear						Placebo group^a^	Placebo vs control ratings	0.63	1, 39	0.43 (0.47)	0.02
	Rating	0.11	1, 39	0.75 (0.29)	0.003	Nocebo group^a^	Nocebo vs control ratings	3.30	1, 39	0.08 (0.70)	0.08
	Experimental condition	2.90	2, 39	0.07 (0.99)	0.13	Control group^a^	Orange-control vs blue-control ratings	0.21	1, 39	0.65 (0.55)	0.01
	Experimental condition × Rating	2.02	2, 39	0.15 (0.99)	0.09	Difference between placebo/nocebo vs control stimuli^b^	Placebo vs control group	0.06	1, 39	0.81 (0.14)	0.001
							Nocebo vs control group	2.58	1, 39	0.12 (0.45)	0.06

^a^ Within-group comparisons.

^b^ Between-group comparisons.

**Fig 4:**
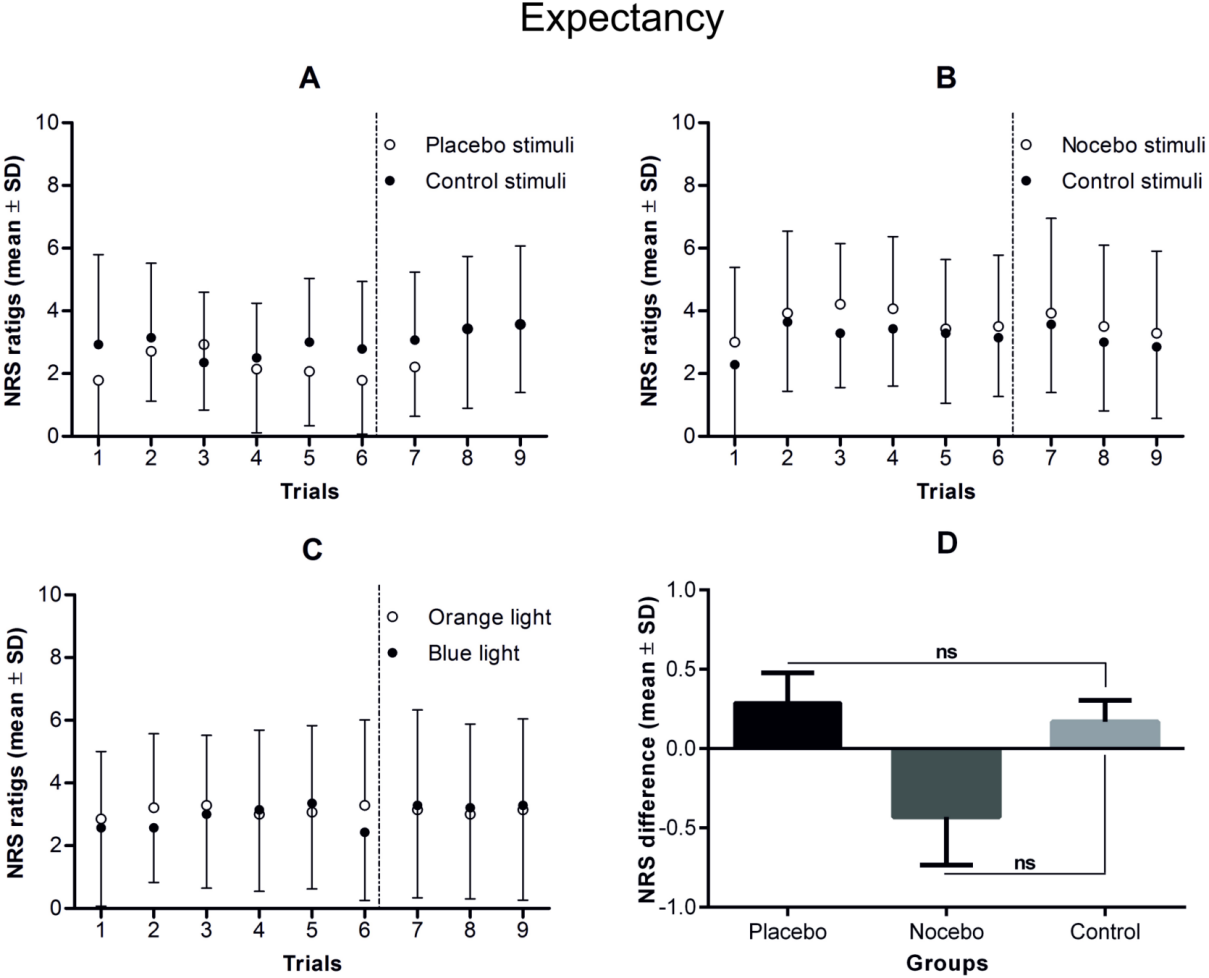
**Expectancy ratings during the conditioning and testing phases in the placebo (A), nocebo (B) and control group (C).** Note that although the conditioning phase consisted of 72 electrical stimuli, which were divided into 4 blocks of 18 stimuli, the participants rated expectancy in only one-third of the trials during the first and the third blocks of stimuli. In the testing phase of the study (separated by vertical dotted lines), consisting of 24 control stimuli, expectancy was rated 6 times. The mean differences in expectancy during the testing phase of the study in each of the study groups are presented in part D of the figure. There were no statistically significant differences in expectancy either within or between the study groups in the testing phase.

**Fig 5:**
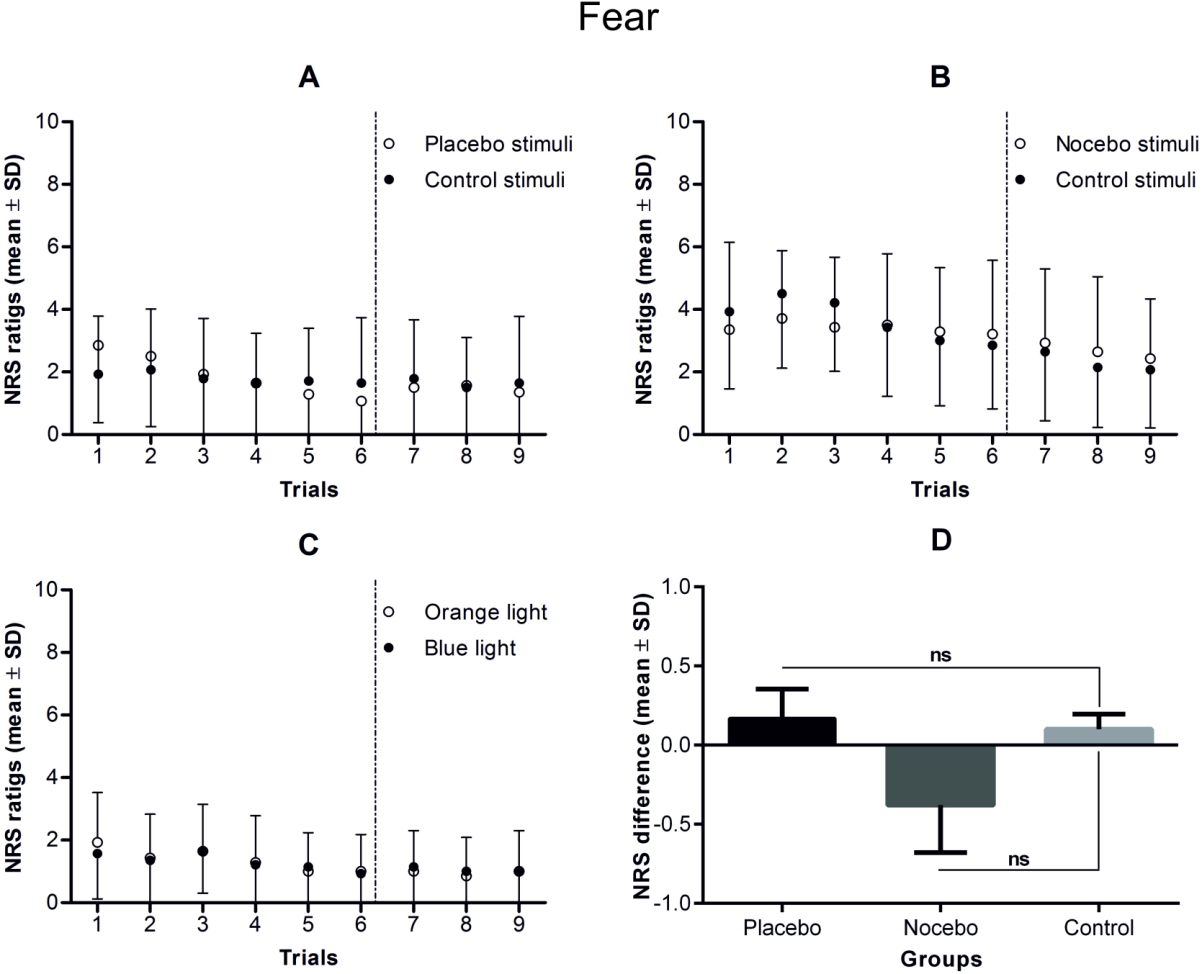
**Fear ratings during the conditioning and testing phases in the placebo (A), nocebo (B) and control group (C).** Note that although the conditioning phase consisted of 72 electrical stimuli, which were divided into 4 blocks of 18 stimuli, the participants rated fear in only one-third of the trials during the first and the third blocks of stimuli. Fear was rated 6 times during the testing phase of the study (separated by vertical dotted lines), which consisted of 24 control stimuli. The mean differences in fear during the testing phase of the study in each of the study groups are presented in part D of the figure. There were no statistically significant differences in fear either within or between study groups in the testing phase.

**Fig 6:**
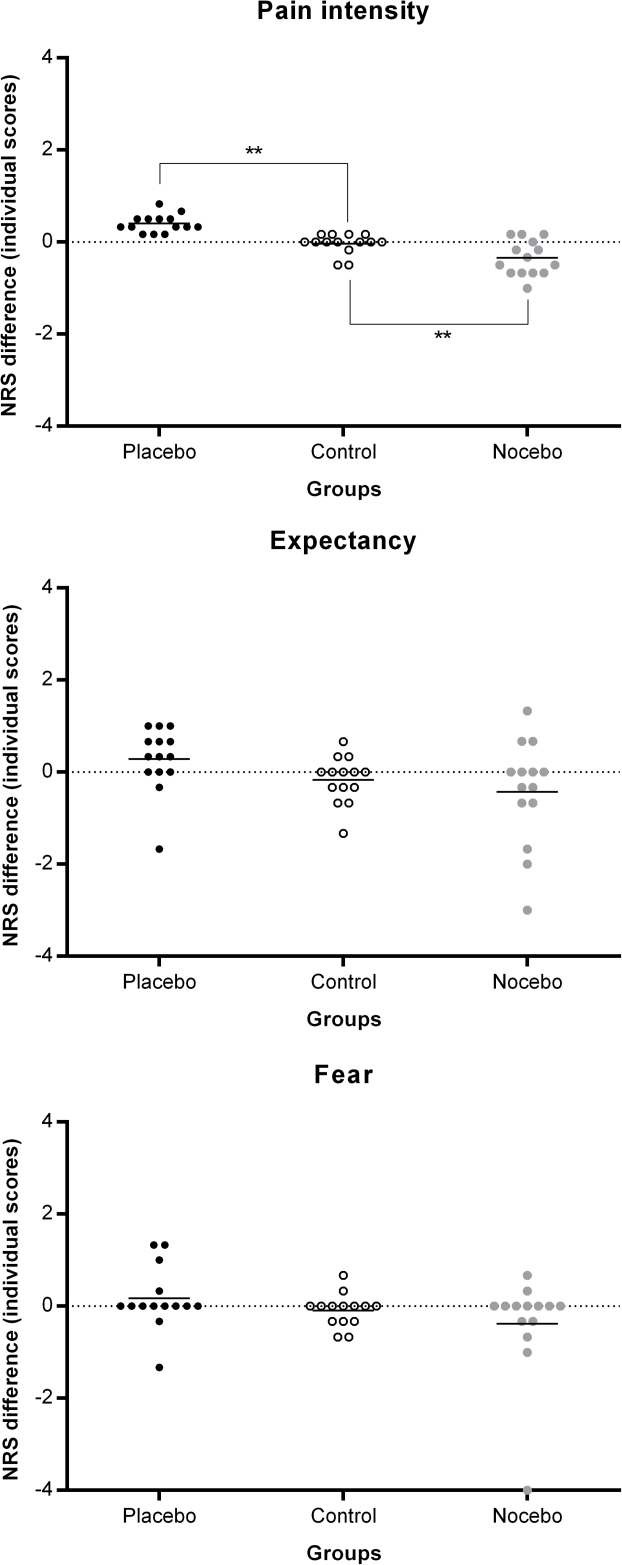
Mean differences (horizontal bars) in pain intensity, expectancy and fear during the testing phase of the study. Individual scores were plotted to show data distribution. In the testing phase, the differences in pain intensity in the placebo and nocebo groups were significantly higher than in the control group. * *p* < 0.01, ** *p* < 0.001.

ANOVAs on the expectancy and fear ratings revealed no statistically significant main effects or interactions (experimental condition × rating), indicating that conditioning had no effect on those variables (see Figs 4–6 and Table 4).

### Regression analyses

Moreover, none of the three regression models predicting the difference in pain intensity of the blue- and orange-associated stimuli in the testing phase of the study was found to be statistically significant, indicating that neither the difference in expectancies for blue- and orange-associated stimuli, nor the difference in fear for blue- and orange-associated stimuli, were able to predict the difference in pain intensity of blue- and orange-associated stimuli in any of the groups (see Table 5).

**Table 5:** Results of separate stepwise multiple regression analyses performed on each group, with the difference in pain intensity of placebo/nocebo- and control-associated stimuli as the dependent variable and differences in the expectancies for and the fear of blue- and orange-associated stimuli as independent variables.

Group	n = 14	Variable	*B*	*T*	*p (power)*	COR *R*^*2*^	*F*	*p*
Placebo	Step 1	Fear	0.48	1.91	0.08 (0.24)	0.17	3.64	0.08
Nocebo	Step 1	Expectancy	0.40	1.52	0.15 (0.14)	0.09	2.32	0.15
Control	Step 1	Expectancy	- 0.33	- 1.22	0.25 (0.09)	0.04	1.48	0.25

## Discussion

Our study found that both placebo analgesia and nocebo hyperalgesia can be induced by classical conditioning without explicit verbal suggestions about analgesia and hyperalgesia, respectively. Moreover, we did not find evidence that self-reported expectancy of pain intensity predicts placebo and nocebo effects when participants experience pain changes during conditioning, but are not given an explicit verbal suggestion about pain modulation. Similarly, self-reported fear was not found to predict either placebo analgesia or nocebo hyperalgesia.

Both placebo analgesia and nocebo hyperalgesia were induced by classical conditioning without any verbal suggestions about decreases or increases in pain, respectively, and without any rituals, such as spreading fake cream, attaching sham TENS electrodes or giving a sugar pill. This result agrees with the previous findings showing that the experience of different pain levels during a conditioning procedure produces placebo analgesia [1,11–13]. However, to the best of our knowledge, nocebo hyperalgesia has not been induced previously by conditioning without verbal suggestions, although there was at least one such attempt [21].

Self-reported expectancy of pain intensity was not found to predict individual pain changes. Among the few previous studies in which classical conditioning without verbal suggestions was sufficient to induce placebo analgesia [1,11–13], just two of them had participants perform expectancy ratings [11,13]. Voudouris and collaborators [13] measured expectancy only before the pretest, so it was impossible to answer the question whether conditioning changed expectancies over time. De Jong and collaborators [11] rated expectancy before each series of pain stimuli but the findings were inconsistent.

Although the effects of classical conditioning are theoretically considered to be mediated by expectancies [31–33], our findings show that this may be not always the case. While the effects of classical conditioning on placebo analgesia induced by verbal suggestions are likely to be mediated by expectancies [5,6], the exposure to distinct pain intensities during conditioning in the present study was sufficient to induce placebo and nocebo effects that were not found to be predicted by trial-by-trial self-reported ratings of expectancy. These findings are in line with the fact that, in some cases, conditioning represents an automatic process which is not mediated by cognitive expectancy [34]. Our results support a model postulating that placebo effects can be learned either consciously or unconsciously, depending on the specific circumstances [14].

Our findings also align with recent findings on placebo analgesia and nocebo hyperalgesia induced by subliminal cues, without prompting participants to expect pain changes [23,24]. In this paradigm, clearly recognizable visual cues were first paired with pain stimuli. Conditioning was followed by a testing phase during which the same conditioned visual cues were presented subliminally. Pain stimuli preceded by subliminally presented conditioned visual cues were rated as more or less painful than control pain stimuli that were not preceded by visual cues, indicating that placebo and nocebo effects were induced without awareness [23,24]. The induction of placebo effects by conditioned stimuli presented subliminally suggests that the effects of conditioning may not involve explicit expectancy, which is consistent with the results of this study.

It should be noted that our findings do not exclude the overall role of expectancy in inducing placebo and nocebo effects. Self-reported explicit expectancy ratings, as measures of “conscious, conceptual belief about the future occurrence of an event” [7, p. 406], may not always predict placebo and nocebo effects. Rather, pre-cognitive associations, defined as “links between events and/or objects that exist outside conscious awareness” [7, p. 411], may be created through hidden conditioning procedures or innate associations that elicit a conditioned response. In summary, expectancy may be either conscious or unconscious [35,36]. Thus, although we did not find evidence for the involvement of self-reported expectancy in the placebo and nocebo effects induced by hidden conditioning, pre-cognitive associations between coloured lights and the level of pain intensity may have been critical in eliciting the observed placebo and nocebo effects. Our results supplement Miller and Colloca’s learning model of the formation of the placebo effect [37,38]. In that model, placebo effects result from expectancies acquired by decoding information from the psychosocial context that includes conditioned stimuli. The current findings suggest that the effects of conditioned stimuli may not always be mediated by self-reported explicit expectancy, but it does not exclude the possibility that they may be mediated by pre-cognitive associations.

Moreover, we did not find evidence that self-reported fear predicts placebo analgesia and nocebo hyperalgesia induced by hidden conditioning. However, this does not exclude the possibility that fear may moderate placebo and nocebo effects, e.g. a previous study showed that fear and stress can eliminate placebo effects induced by verbal suggestions [25]. We only found that trial-by-trial fear ratings do not predict the magnitude of placebo analgesia and nocebo hyperalgesia when pain intensity changes are experienced without any outcome-directed warning or information. Fear has been described as an emotional response elicited by an identifiable and explicit threatening stimulus [39,40]. In other words, fear emerges with subjective *certainty* that an aversive stimulus is impending and occurs in situations of certain threat and is manifested by heightened attention to specific threat stimuli [41,42]. As no explicit verbal suggestions were given in our study and self-reported expectancy did not predict placebo effects, our participants might not have been certain that a specific colour preceded more pain. Thus, no evidence was found for the involvement of fear in the formation of the placebo analgesia and nocebo hyperalgesia induced by hidden conditioning without explicit verbal suggestions. This finding, together with the results showing that expectancy did not predict either placebo analgesia or nocebo hyperalgesia, suggests that hidden conditioning may be a distinct mechanism producing placebo and nocebo effects, and that neither expectancy nor fear might be involved in the formation of such effects induced without verbal suggestions.

Some limitations of our study should be acknowledged. First, only females participated in the study, and in light of sex differences in pain perception [43,44], the results may not be generalisable to men. On the other hand, as only women participated in many of the previous studies on the mechanisms of placebo effects [11,15,17,22,45,46], the results of our study can be directly compared to previous research. Second, the sample size was rather small but it was based on previous studies that used a similar ‘n’ and had shown significant effects (14 persons in each group) in similar paradigms [15–17,22,26]. Moreover, power values together with effect sizes suggest that the sample size was enough to find statistically significant results (Tables 4 and 5).

Our results have implications for both pain research and medical practice. Caution should be used in considering self-reported expectancy of pain intensity as predictors of placebo and nocebo effects, as pain changes can occur even when participants are not fully aware of the ongoing treatment and anticipated outcomes. Brain imaging studies might provide evidence of more implicit mechanisms related to the formation of placebo and nocebo effects induced by classical conditioning without explicit verbal suggestions, e.g. pre-cognitive associations.

The results of the current study suggest that pain is a complex phenomenon. It can become worse or better after repetitive negative or positive experiences that are associated with the colour of the treatment and other uncontrolled conditioning cues. Thus, environmental factors associated with pain experience may serve as conditioned cues producing symptom worsening or improvement even if explicit instructions are not given and, presumably, cognitive expectancies play no role.
